# Coronary artery spasm following mannitol administration during partial nephrectomy: A case report

**DOI:** 10.1097/MD.0000000000043527

**Published:** 2025-08-01

**Authors:** Jae-Myung Kim, Mi Roung Jun

**Affiliations:** a Department of Surgery, Gyeongsang National University College of Medicine, Gyeongsang National University Hospital, Jinju, Korea; b Department of Anesthesiology and Pain Medicine, Gyeongsang National University College of Medicine, Gyeongsang National University Hospital, Jinju, Korea.

**Keywords:** case report, coronary vasospasm, mannitol, variant angina, ventricular fibrillation

## Abstract

**Rationale:**

Coronary vasospasm (CAS) is often triggered by autonomic imbalance and can lead to severe cardiac events, particularly in patients with undiagnosed variant angina. Mannitol, commonly used for renal protection, may inadvertently activate the sympathetic nervous system, potentially leading to CAS in susceptible patients. This rare case highlights the potential cardiac risks of mannitol-induced CAS during robotic-assisted surgery.

**Patient concerns:**

A 66-year-old male with no known cardiac history underwent robotic-assisted partial nephrectomy. Approximately 40 minutes after receiving a high-dose mannitol infusion (75 g), he developed frequent ventricular premature contractions followed by sudden ventricular fibrillation.

**Diagnoses:**

During the operation, post-resuscitation transthoracic echocardiography showed regional wall motion abnormalities. After the operation, emergent coronary angiography revealed no significant stenosis. A coronary spasm provocation test performed 10 days later confirmed the diagnosis of variant angina due to significant CAS.

**Interventions:**

In the operating room, prompt cardiopulmonary resuscitation and 3 rounds of defibrillation successfully restored circulation. Following emergent transthoracic echocardiography and coronary angiography, the patient was admitted to the intensive care unit for further evaluation and management.

**Outcomes:**

The patient recovered without neurologic sequelae. Calcium channel blocker therapy was initiated, and regular follow-up was arranged at the outpatient department.

**Lessons:**

This case underscores the potential cardiac risks associated with high-dose mannitol in patients with coronary hyperreactivity, highlighting the importance of personalized perioperative management and close cardiovascular monitoring. In high-risk patients, alternative strategies for renal protection should be considered to mitigate the risk of life-threatening cardiac events.

## 1. Introduction

Coronary vasospasm (coronary artery spasm [CAS]) typically presents as recurrent angina at rest, often accompanied by ST-segment changes on an electrocardiogram (ECG).^[1]^ Diagnosing variant angina preoperatively is challenging, particularly in asymptomatic patients with normal assessments. Lanza et al^[2]^ have emphasized that autonomic dysregulation, involving both sympathetic and parasympathetic activity, plays a key role in CAS pathogenesis. Research further indicates that sympathetic nervous system activation can precede CAS episodes by mere minutes.^[3]^

Although mannitol is commonly used for renal protection in the perioperative setting, its efficacy in preventing acute renal failure is debated.^[4]^ Mannitol initially causes volume expansion and left ventricular end-diastolic pressure elevation, followed by subsequent hypovolemia. This hypovolemia may activate the sympathetic nervous system, increasing the risk of CAS in susceptible individuals.^[5]^

We present a rare case of fatal ventricular fibrillation, likely caused by CAS, following high-dose mannitol infusion during a robotic-assisted partial nephrectomy. To our knowledge, this is one of the first reported cases of mannitol-induced CAS occurring during robotic-assisted surgery, emphasizing the need for heightened awareness of its potential cardiac risks.

## 2. Patient information

A 66-year-old man (height: 169 cm and weight: 51 kg) was scheduled for a robotic-assisted partial nephrectomy after a 2.5 cm predominantly cystic mass with focal solid components was incidentally detected in the lower pole of the right kidney on routine abdominal CT scan performed during a general health checkup. The lesion was considered suspicious for malignancy, and nephron-sparing surgery was indicated. His medical history was unremarkable except for a left inguinal hernia repair under spinal anesthesia at an outpatient clinic a few months earlier.

## 3. Clinical findings and therapeutic intervention

**Figure 1.**
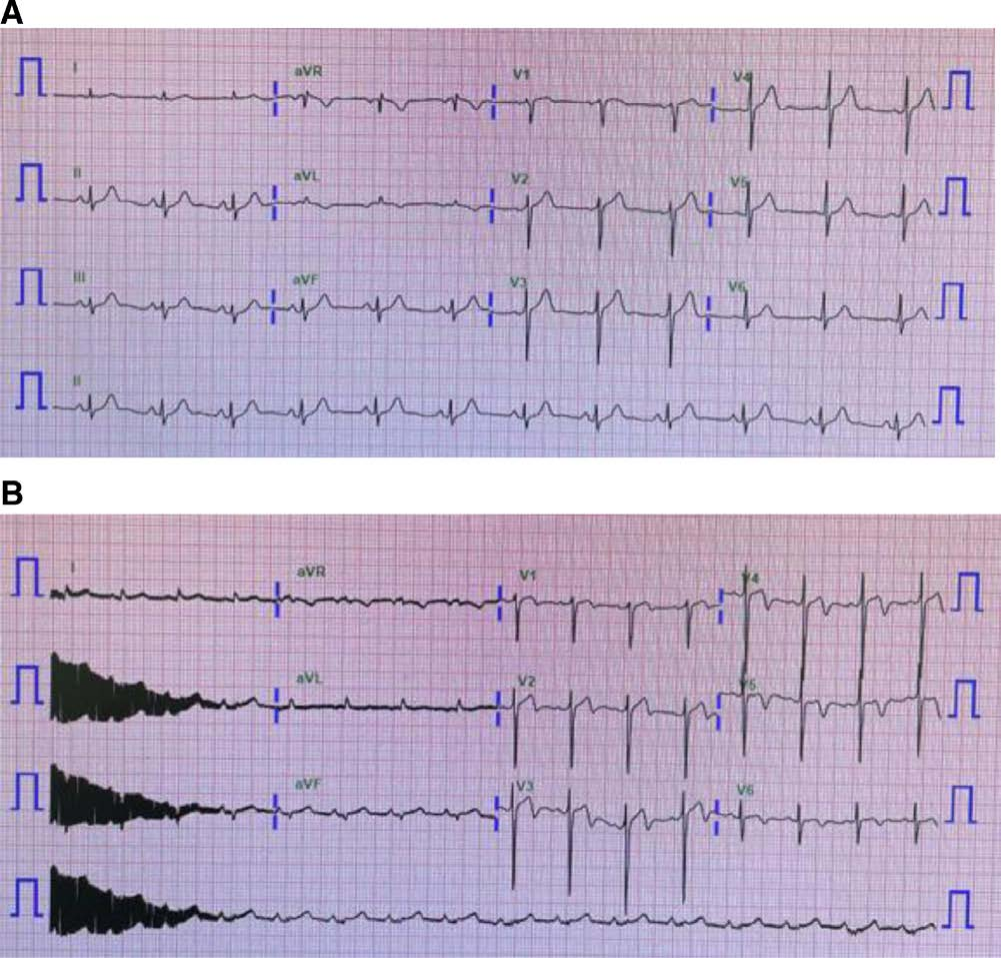
(A) Preoperative electrocardiogram and (B) Post-resuscitation electrocardiogram showing T wave inversions in leads I, aVL, and V2–V6, consistent with transient ischemia, possibly involving the lateral territory, or postarrest changes. aVF = augmented voltage foot, aVL = augmented voltage left arm, aVR = augmented voltage right arm.

Preoperative assessments revealed a normal sinus rhythm with no significant findings on a 12-lead ECG (Fig. 1A). Two-dimensional echocardiography showed a left ventricular ejection fraction of 81% with no significant abnormalities. Chest radiography was also unremarkable. Preoperative laboratory evaluations indicated normal renal and hepatic function, with serum creatinine level of 1.0 mg/dL, estimated glomerular filtration rate of >60 mL/min/1.73 m^2^, AST of 17 U/L, and ALT of 17 U/L. The patient’s vital signs were stable, with a blood pressure of 140/86 mm Hg, heart rate of 88 beats/min, and body temperature of 36.7℃. physical examination revealed no notable abnormalities.

On the day of surgery, the patient received 0.2 mg of intramuscular glycopyrrolate for premedication, and intravenous access was established. Upon arrival in the operating room (OR), routine monitoring showed an initial blood pressure of 108/64 mm Hg, heart rate of 98 bpm, and oxygen saturation of 100%. The ECG was normal except for mild tachycardia. General anesthesia was induced with lidocaine (40 mg), propofol (120 mg), fentanyl (50 mcg), and rocuronium bromide (50 mg). Endotracheal intubation was uneventful.

The patient was then repositioned from supine to the left lateral decubitus position for surgery. Approximately 10 minutes later, the da Vinci Single Port™ Surgical System was docked, and the surgery proceeded without complications. Anesthesia was maintained with desflurane at 4.83 to 5.08 minimum alveolar concentration in a 50:50 air–oxygen mixture, with the bispectral index maintained between 40 and 60. Before clamping the renal arteries, the urologist requested an infusion of 75 g of mannitol (1.47 g/kg), which was administered over 25 minutes, a standard practice at our institution.

Approximately 40 minutes after the mannitol infusion was completed, the ECG showed ventricular premature contractions (VPCs). Eleven minutes after the onset of VPCs, the rhythm progressed to ventricular fibrillation. An immediate call for assistance was made, and the patient was repositioned to the supine position. Cardiopulmonary resuscitation was initiated, along with arterial line placement and large-bore venous access. Initial blood gas analysis revealed a pH of 7.15, PCO_2_ of 64 mm Hg, PO_2_ of 116 mm Hg, sodium (Na) of 132 mmol/L, potassium (K) of 4.6 mmol/L, and a base excess of −6.6 mmol/L. Epinephrine (1 mg) was administered every 3 minutes. Three rounds of defibrillation, with energy levels between 150 and 200 J, successfully restored cardiac rhythm. Following the return of spontaneous circulation, infusions of nitroglycerin (5 mcg/kg/min) and norepinephrine (0.15–0.18 mcg/kg /min) were initiated to maintain a mean arterial pressure above 60 mm Hg.

As the surgical procedure neared completion, the surgeon proceeded with primary closure of the incision. Simultaneously, a cardiologist performed an emergency transthoracic echocardiogram (TTE) in the OR, revealing regional wall motion abnormalities throughout the left ventricle. The patient was subsequently transferred to the percutaneous coronary intervention unit, where coronary angiography showed no significant stenosis. A coronary spasm provocation test was deferred due to the ongoing nitroglycerin infusion during cardiopulmonary resuscitation, which could interfere with the test result. Following the procedure, the patient was transferred to the intensive care unit.

## 4. Follow-up and outcomes

**Figure 2.**
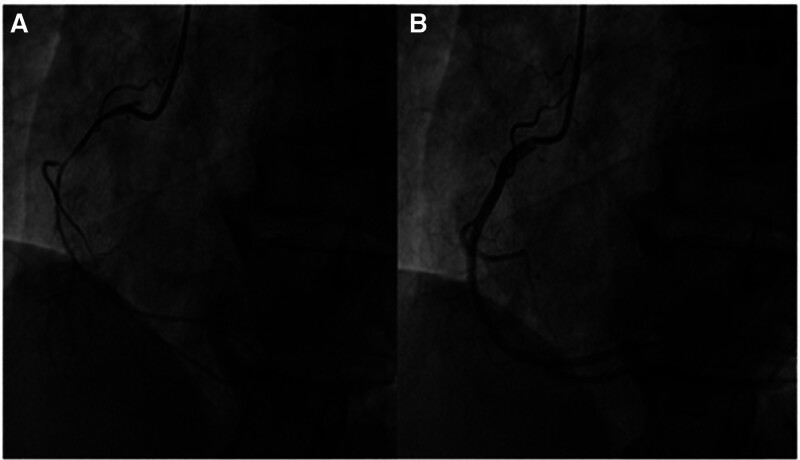
Coronary angiography of a patient with coronary vasospasm. (A) Ergonovine-induced spasm in the right coronary artery (RCA) during a provocation test. (B) The vasospasm was resolved following administration of nitroglycerin (NTG).

A post-resuscitation ECG was obtained (Fig. 1B), and TTE performed the following day revealed an ejection fraction of 58% with grade 2 diastolic dysfunction, likely reflecting transient myocardial dysfunction secondary ventricular fibrillation and cardiac arrest requiring resuscitation. Ten days later, an invasive coronary spasm provocation test demonstrated a significant positive spasm of the right coronary artery (Fig. 2). The patient was diagnosed with variant angina, and the cardiologist recommended lifelong treatment with a non-dihydropyridine calcium channel blocker and nicorandil.

## 5. Discussion

The clinical case highlights a life-threatening CAS in a patient with undiagnosed variant angina, leading to VPCs followed by ventricular fibrillation. During the preoperative evaluation, the patient exhibited no clinical signs of chest pain, and diagnostic tests, including ECG and echocardiography, revealed no significant cardiac abnormalities. However, before discharge, the patient reported experiencing occasional episodes of chest tightness upon waking at dawn, which he had not previously considered concerning.

CAS is characterized by a sudden, reversible, and significant narrowing of an epicardial coronary artery, which can lead to partial or total occlusion, resulting in impaired myocardial perfusion.^[2]^ This phenomenon occurs due to spontaneous vascular smooth muscle overactivity and increased vascular wall tension. While CAS is associated with various coronary syndromes, it is predominantly identified as the primary cause of variant angina.^[2]^ The exact pathophysiology of CAS remains unclear. CAS can also occur in the perioperative period. Koshiba and Hoka^[6]^ identified multiple perioperative factors that may precipitate coronary spasm, including inadequate depth of general anesthesia with inhalational agents, vasopressor administration, vagal nerve stimulation, and epidural anesthesia. Their study also found that CAS frequently occurred during surgeries, especially, those involving the upper and lower abdominal regions.

Mannitol, a low-molecular-weight sugar alcohol, is widely used as an osmotic diuretic and free radical scavenger.^[7,8]^ It has been shown to reduce cellular swelling, improve renal function after hypoxic injury, and protect against nephron loss in animal models.^[8,9]^ Consequently, mannitol, typically administered in doses ranging from 12 to 50 g, is a crucial renal protective agent during partial nephrectomies and kidney transplantations.^[7,10]^ However, recent studies have questioned its role in renal protection and its effectiveness in reducing complications during kidney surgeries.^[11,12]^ In this case, the urologist recommended the infusion of 75 g of mannitol, which exceeds 1 g/kg – a high dose compared to those evaluated in prior studies. Mannitol doses exceeding 1 g/kg have not demonstrated additional benefits in preventing renal injury.^[10]^ Moreover, high doses have been associated with excessive fluid shifts and electrolyte imbalances, particularly affecting potassium levels.^[13]^

Mannitol exerts a rapid intravascular volume-enhancing effect within 4 to 5 minutes of administration, reaching peak effects in approximately 30 to 45 minutes.^[13,14]^ This expansion in intravascular volume can significantly alter hemodynamic parameters, including left ventricular preload, cardiac output, and afterload.^[14]^ Additionally, mannitol administration has been linked to reduced coronary blood flow and an increased myocardial oxygen demand due to the elevation of left ventricular end-diastolic pressure.^[14]^ As mannitol administration progresses, it ultimately induces intravascular volume depletion.^[5]^ These combined effects can trigger sympathetic activation, which initially causes coronary vasodilation and improves myocardial blood flow.^[15]^ However, excessive sympathetic outflow has been linked to CASs, particularly in patients with hyperactivity coronary segments.^[2]^ In this case, the fatal arrhythmia occurred approximately 40 minutes after the completion of mannitol infusion, aligning with its peak hemodynamic effects and potential sympathetic surge. Given the patient’s history of coronary artery hyperreactivity, as demonstrated by a positive coronary reactivity test, this condition may have predisposed him to CAS triggered by sympathetic overactivity.

To ensure a comprehensive evaluation, several differential diagnoses for intraoperative ventricular fibrillation and cardiac arrest were considered, including electrolyte abnormalities, structural heart disease, and acute coronary syndrome. However, these were deemed unlikely based on normal pre- and postoperative laboratory values, unremarkable echocardiography results. Although ischemic changes were observed on emergency TTE in the OR and on the ECG following resuscitation, subsequent coronary angiography revealed no significant coronary artery stenosis. Therefore, we concluded that CAS, potentially triggered by mannitol-induced volume depletion and excessive sympathetic activation, was the most plausible etiology.

In conclusion, this case suggests a correlation between CAS and high-dose mannitol administration during nephron-sparing surgery. Despite an unremarkable preoperative cardiac evaluation, the patient’s undiagnosed variant angina, characterized by occasional chest tightness, may have predisposed him to a life-threatening ventricular arrhythmia. While mannitol is commonly used for renal protection, its potential to exacerbate sympathetic activation may have triggered CAS in this patient. This case underscores the importance of thorough medical history-taking to identify subtle signs of cardiac abnormalities and highlights the need for careful dose management of mannitol to prevent hemodynamic and electrolyte disturbances. Moving forward, greater awareness of CAS risk factors, along with individualized perioperative management, may help prevent similar adverse events in at risk patients, particularly when using agents like mannitol that can induce hemodynamic imbalances. However, given the single-case nature of this report and the potential influence of other perioperative factors, a direct causal relationship cannot be definitively established. Future studies are needed to further investigate the impact of mannitol on coronary reactivity and explore alternative renal protective strategies that minimize the risk of hemodynamic disturbances.

## Acknowledgments

The authors appreciate the contributions of the medical teams involved in the patient’s care.

## Author contributions

**Conceptualization:** Jae-Myung Kim, Mi Roung Jun.

**Supervision:** Mi Roung Jun.

**Writing** – **original draft:** Jae-Myung Kim.

**Writing** – **review & editing:** Mi Roung Jun.
